# Differentiating induced versus spontaneous subduction initiation using thermomechanical models and metamorphic soles

**DOI:** 10.1038/s41467-021-24896-x

**Published:** 2021-07-30

**Authors:** Xin Zhou, Ikuko Wada

**Affiliations:** Department of Earth and Environmental Sciences, University of Minnesota, Minneapolis, MN USA

**Keywords:** Geodynamics, Structural geology, Geophysics

## Abstract

Despite the critical role of subduction in plate tectonics, the dynamics of its initiation remains unclear. High-temperature low-pressure metamorphic soles are vestiges of subduction initiation, providing records of the pressure and temperature conditions along the subducting slab surface during subduction initiation that can possibly differentiate the two end-member subduction initiation modes: spontaneous and induced. Here, using numerical models, we show that the slab surface temperature reaches 800–900 °C at ~1 GPa over a wide range of parameter values for spontaneous subduction initiation whereas for induced subduction initiation, such conditions can be reached only if the age of the overriding plate is <5 Ma. These modeling results indicate that spontaneous subduction initiation would be more favorable for creating high-temperature conditions. However, the synthesis of our modeling results and geological observations indicate that the majority of the metamorphic soles likely formed during induced subduction initiation that involved a young overriding plate.

## Abstract (web summary)

The mechanism of subduction initiation is a key to modern plate tectonics. Here, using numerical modeling and geological observations, the authors find that the majority of active and paleo subduction zones with metamorphic soles likely formed during induced subduction initiation that involved a young overriding plate.

## Introduction

Subduction initiation (SI) holds a key to the inception of plate tectonics and the evolution of the solid Earth^1–6^. SI is also an important factor that impacts the motion and the configuration of tectonic plates; for example, the SI events that lead to the formation of the Izu–Bonin–Mariana (IBM) subduction zone could have resulted in major changes in the Pacific plate motion^7,8^. During SI, the downward motion of the cold oceanic lithosphere and the asthenospheric mantle flow that is induced by the subducting slab lead to a rapid evolution of the thermal and mechanical structures of the region^9,10^. However, how subduction initiates in the framework of plate tectonics remains elusive, resulting in large uncertainties in the thermal evolution of tectonic plates and the dynamics of mantle flow during SI.

**Fig. 1:**
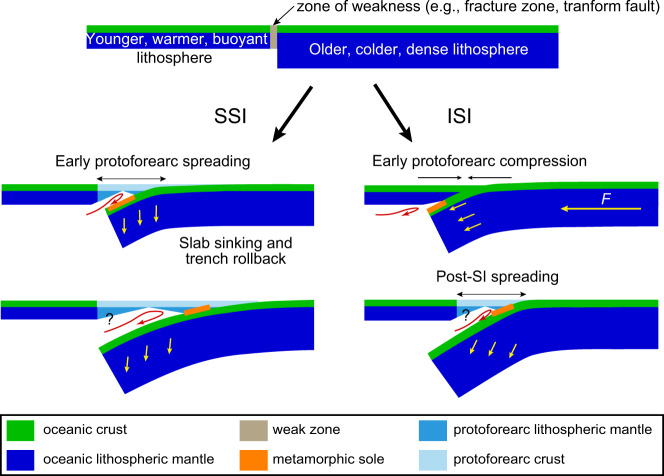
Spontaneous subduction initiation and induced subduction initiation in an intra-oceanic setting. The schematic diagram highlights the difference in the relative timing of the formation of metamorphic sole and proto-forearc spreading in the two modes (modified from refs. ^15,20,85^).

Two end-member SI modes have been proposed based on geological observations and geodynamic modeling: spontaneous and induced SI (SSI and ISI, respectively)^11–17^ (Fig. 1). SSI and ISI are also referred to as vertically-forced and horizontally-forced SI, respectively, in recent research^18^. SSI occurs when the negative buoyancy of a plate can no longer be supported by resisting forces, such as the coupling force with the neighboring less dense plate^19,20^. SSI results in subsidence, trench retreat, and extensional magmatism, forming proto-forearc oceanic basins^14,15,21–23^. ISI occurs when a plate experiences external tectonic forces and is forced to subduct beneath another plate, resulting in compressive uplift of the proto-forearc through a series of thrust faulting^11,24–26^. Subsequent rollback of the subducting slab may allow extensional magmatism and formation of a proto-forearc basin, resulting in a similar lithological architecture to ISI^15^.

One of the possible clues to differentiating the two SI modes for active and paleo subduction zones is metamorphic soles, which are thin sheets (a few to several hundred meters thick) of highly deformed high-temperature low-pressure (HT-LP) metamorphic rocks. They represent oceanic crust that were scraped off from the downing going slab during SI, welded to the base of the overriding forearc lithosphere, and exhumed together as supra-subduction zone (SSZ) ophiolite complexes^27–32^. In this theoretical framework, if SI occurs spontaneously, the metamorphic soles should approximately be coeval with the ophiolites in SSI, but in ISI, the metamorphic soles should predate the ophiolites^15^ (Fig. 1).

**Fig. 2:**
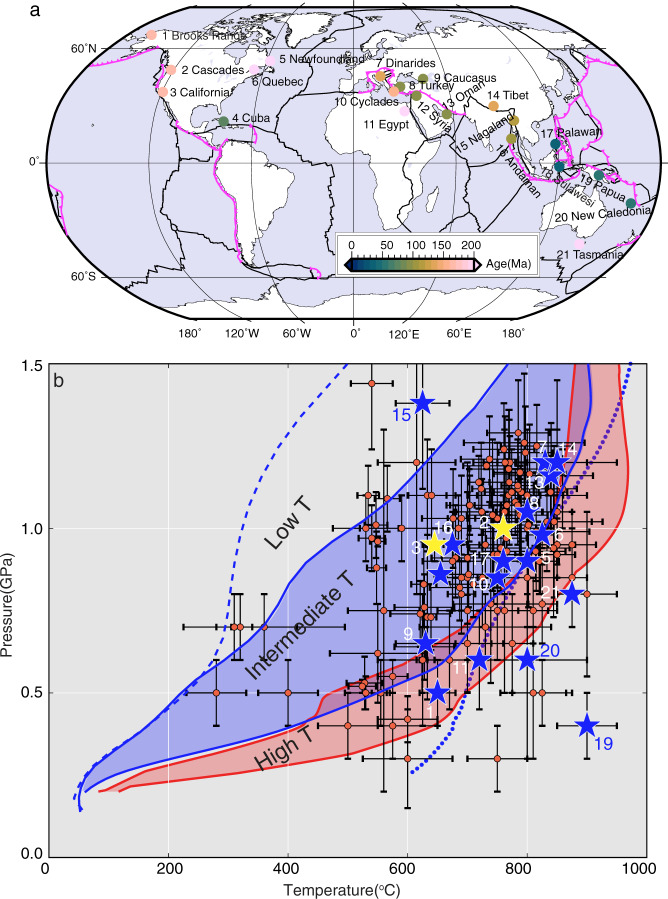
Distribution and thermobarometry data of metamorphic soles. **a** Localities of metamorphic soles (colored circles, updated from ref. ^30^) and **b** their pressure–temperature conditions (red circles). The color of the circles in **a** indicates the age of the metamorphic soles, which is interpreted to be equivalent to the approximated timing of subduction initiation. In **b**, blue and yellow stars indicate the peak T condition for a given locality that is likely to have experienced SI in intra-oceanic and passive margin settings, respectively. The black error bars indicate the pressure and temperature evaluation errors. Red region (high-temperature region) outlined by red solid lines indicates the ranges of PT paths predicted by our SSI models with 1–20 Ma overriding plate, and blue region (intermediate temperature region) outlined by blue solid lines indicate the ranges of PT paths predicted by our ISI models with 1–5 Ma overriding plate. Dotted and dashed blue line indicates the PT paths of an ISI model with a 0 and 40 Ma overriding plate, respectively. The numbers in **a** and **b** indicate the localities: 1 – Brooks Range; 2 – Cascadia; 3 – California; 4 – Cuba; 5 – Newfoundland; 6 – Quebec; 7 – Dinarides; 8 – Turkey; 9 – Caucasus; 10 – Cyclades; 11 – Egypt; 12 – Syria; 13 – Oman; 14 – Tibet; 15 – Nagaland; 16 – Andaman; 17 – Palawan; 18 – Sulawesi; 19 – Papua; 20 – New Caledonia; 21 – Tasmania. Data and relevant references are summarized in Supplementary Table 1.

In this study, using 2-D thermomechanical models, we quantify the slab surface temperatures during the two SI modes in intra-oceanic settings and assess which SI mode explains the PT records of metamorphic soles better. Intra-oceanic subduction may initiate at transform faults, fracture zones, mid-ocean ridges, back-arc spreading centers, former oceanic detachment faults, and fossil subduction margins^8,18,22,33–36^. It has been suggested that subduction may also initiate at passive continental margins through varying weakening mechanisms, such as sedimentary loading^37^, pressure gradients arising from density differences^38^, and grain damage^39^. However, the observations indicate that SI at passive margins has been rare in the last 100 Ma, and most SI events occurred near or at pre-existing weak zones^18^. We compile PT estimates of metamorphic soles from 21 localities of varying tectonic settings (Fig. 2). The peak temperatures are as high as 850–900 °C at shallow depths (0.3–1.3 GPa, approximately equivalent to 10–40 km depth)^40^. The exact position of metamorphic soles relative to the slab surface is unclear in most cases. If they were situated deeper than the slab surface, the slab surface temperature would have been even hotter than the temperature indicated by the metamorphic soles^30^. While our modeling results indicate that SSI can lead to high-temperature conditions that are comparable to the PT records of metamorphic soles over a wide ranges of parameter values, the synthesis of the PT records, regional geological observations, and the modeling results indicate that ISI is a more common mode of SI that produces metamorphic soles.

## Results

### Numerical model setup

**Fig. 3:**
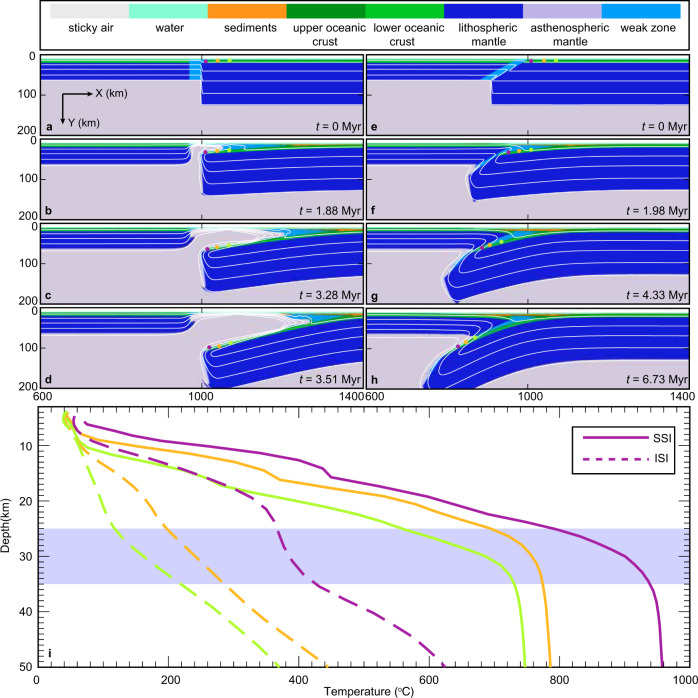
Thermal evolution of incipient subduction zones. **a**–**d** Evolution of spontaneous subduction initiation (SSI), **e**–**h** evolution of induced subduction initiation (ISI), and **i** P-T paths of tracers along the slab surface at 10 km (purple), 30 km (orange), and 60 km (green) distance from the slab tip. The approximate locations of the tracers are indicated by three colored circles in **a**–**h**. The depth range where metamorphic soles are typically formed is highlighted in light blue in **i**.

We develop a series of thermomechanical models for intra-oceanic SI (see Methods). The model consists of two lithologically layered oceanic plates, a weak zone between the plates, and the asthenospheric mantle (Fig. 3 and Supplementary Fig. 1; “Methods” section). In the entire model domain, visco-plastic rheology is applied. Rheological parameters, as well as other material properties, vary among different lithologies (“Methods” section and Supplementary Tables 2 and 3). In a pair of models for SSI and ISI that we first present below (hereafter referred to as the reference models; Fig. 3), all model parameters except the geometry of the weak zone and the kinematics of the incoming plate are identical. The weak zone is a vertical column in SSI and a dipping layer in ISI, both with an apparent width at the surface of 30 km. The orientation of the weak zone is chosen to be approximately parallel to the initial motion of the subducting plate, minimizing complex deformation near the plate boundary. The weak zone represents a transform fault, a fracture zone, or any other possible pre-existing weakness in the ocean basin. In the ISI model, the motion of the incoming plate is kinematically prescribed with a convergence rate of 3 cm/yr. In both reference SSI and ISI models, the overriding and subducting plate ages are 20 and 100 Ma, respectively.

### Modeled slab surface temperatures

In the SSI model, as the older slab starts to sink, the material in the weak zone and the hot asthenospheric mantle fill in the forearc space above the slab. This results in a rapid increase in the temperature along the top of the slab (Fig. 3a). The SSI is accompanied by a large forearc extension, forming a proto-forearc basin (Fig. 3a–d). By contrast, in the ISI model, the slab is jammed against the overriding plate, and the compressive stress state in the forearc does not allow the hot asthenosphere to flow into the region above the slab, resulting in a cooler subduction interface (Fig. 3e–h). The slab initially rolls back slightly by ~100 km to accommodate plate deformation but without forming a proto-forearc basin (Fig. 3f). The strong plate coupling results in slower subduction than in the SSI model. With a smaller prescribed velocity of the incoming plate (e.g., 1 cm/yr), however, the sinking rate exceeds the convergence rate, resulting in trench retreat and proto-forearc spreading (Supplementary Fig. 2).

The temperature of the marker closest to the tip of the slab surface increases much more rapidly in SSI than in ISI (red solid and dashed lines in Fig. 3i). This occurs because the upwelling mantle heats up the slab surface in SSI whereas the upwelling mantle is absent in ISI at the beginning. The temperature difference between SSI and ISI peaks around 30 km depth, reaching more than 500 °C. The temperature at the slab surface decreases up-dip away from the slab tip in both modes, and the temperature difference between the two modes at a given depth remains relatively uniform. These results indicate that SSI provides much hotter conditions that are favorable for the formation of metamorphic soles with high peak temperatures except when the overriding plate is extremely young as further discussed below. In both cases, the highest temperature for a given SI style is attained closest to the slab tip, making it the most preferable location to form a metamorphic sole. Hereafter, we approximate the slab tip temperature by the marker that is closest to the slab tip.

**Fig. 4:**
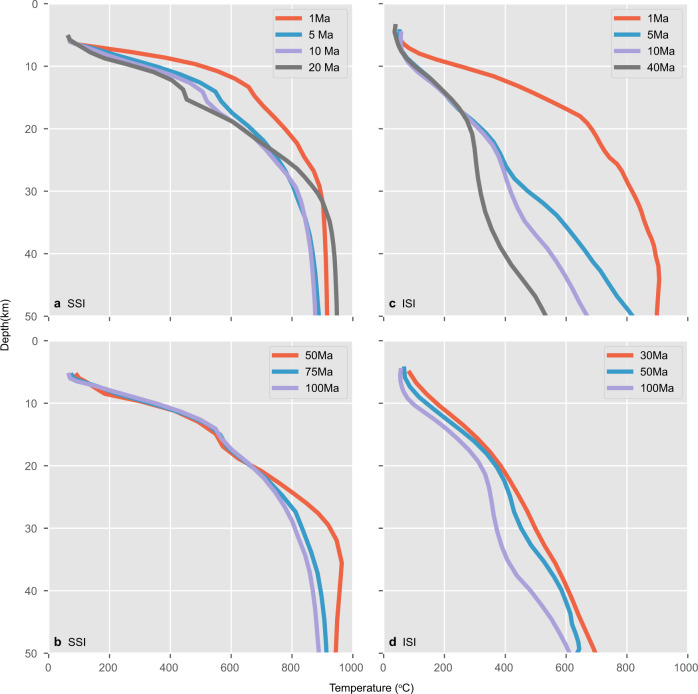
Slab surface pressure–temperature path. Variation in the pressure–temperature path of a tracer along the slab surface at 10 km from the slab tip for (top panels) different overriding plate ages with a 100 Ma subducting plate and (bottom panels) different subducting plate ages with a 20 Ma overriding plate for the case of **a**, **b** spontaneous subduction initiation (SSI) and **c**, **d** induced subduction initiation (ISI), respectively.

The slab surface temperature during SI is impacted by the initial thermal states and thus the ages of the two plates and also by their age contrast as it controls the density contrast and the negative buoyancy of the subducting slab^22^. Unlike ISI, there is a minimum density contrast that needs to be satisfied for SSI to occur. A series of modeling experiments indicate that the ages of the overriding and subducting plates need to be <~20 and >~50 Ma, respectively. Over these ranges of plate ages that provide sufficient density contrast for SSI, the slab surface temperature at shallow depths, particularly at 10–20 km depths, is higher for a younger and thinner overriding plate, which is underlain by hot asthenospheric mantle at shallower depths that heats up the slab more efficiently (Fig. 4a). For the slab age of 100 Ma, the difference in the slab surface temperature between the models with a 1 and 20 Ma overriding plate is more than 200 °C at 15 km depth (Fig. 4a). With a younger overriding plate, the larger density contrast between the two plates causes the faster sinking of the slab and more vigorous mantle upwelling, partially contributing to the warmer slab tip. These warming effects of younger overriding plates are large enough to overshadow the effect of faster downward advection of the slab that causes a cooler slab surface condition at a given depth. At greater depths (>30 km depth), relatively slow subduction in SSI allows the variation in the slab tip temperature to decrease through thermal diffusion. The higher slab surface temperature at >30 km in the model with a 20 Ma overriding plate is caused by even slower subduction due to the smaller density contrast between the two plates. By contrast, the variation in the slab surface temperature with the subducting slab age in SSI is smaller, particularly among older subducting slabs (Fig. 4b). The smaller variation in the slab surface temperature is simply attributed to the fact that the thermal state of tectonic plates (approximately proportional to the square root of plate age) does not change significantly at older ages. The above maximum overriding plate age and minimum slab age for SSI are based on visco-plastic rheology and may differ from those that incorporate the effects of elasticity^11,25,41^. However, because the slab surface temperature during SI is controlled strongly by the upwelling asthenospheric mantle flow and thus the age of the overriding plate, as shown above, the effect of elasticity on the slab surface temperatures during SI is expected to be relatively small.

In ISI, the subducting plate directly abuts against the overriding plate without the upwelling of the hot asthenospheric mantle, and the slab surface temperature is even more sensitive to the overriding plate age than in SSI (Fig. 4c). The subducting slab surface is much cooler than that in SSI for an older overriding plate age. The difference in the slab surface temperature between SSI and ISI, however, diminishes for an extremely young overriding plate (<1 Ma), which must be at or near the spreading center. In such cases, the thermal conditions between SSI and ISI become indistinguishable. The effect of the subducting plate is small (Fig. 4d) as in the case of SSI (Fig. 4b). These results indicate that the slab surface temperature during SI is impacted strongly by the dynamics and the temperature of the overriding plate.

The weak zone between the two plates is necessary for SI in the numerical simulation and represents a collective effect of all weakening mechanisms, such as faulting^12^, fracturing, shear heating^41^, thermal softening^42^, and grain damage^43,44^. The width at which such weakening occurs depends on the exact mechanism and is likely to affect the dynamics of SI. Our modeling results indicate that for a given strength of the weak material, a wider weak zone reduces plate coupling and allows faster subduction in both SSI and ISI. This results in a cooler slab surface temperature due to faster downward advection of the slab in SSI (Supplementary Fig. 3a). In ISI, the subduction rate can increase beyond the convergence rate, resulting in slab roll back. In such a case, the dynamics of the slab and the surrounding mantle is similar to that in SSI in that the slab no longer abuts the overriding plate and the asthenospheric mantle upwells between the two plates. However, the thermal effect of the upwelling mantle on the slab surface is relatively small due to fast subduction, and the slab surface remains colder than in the SSI counterpart (Supplementary Fig. 3b). Thus, the width of the weak zone has a relatively small net effect on the slab surface temperature during ISI whereas, during SSI, the slab surface temperature decreases with increasing width of the weak zone. However, field and seismological observations indicate that the width of weak zones (transform fault/fracture zones) is typically no >30 km^45^. For the width range of <30 km, the slab surface is much hotter during SSI than during ISI.

In our ISI models, the plate convergence is imposed by applying a convergence velocity to the incoming plate. However, the imposed convergence rate is not necessarily equal to the effective subduction rate, which depends also on the buoyancy of the subducting slab. Our modeling results indicate that when the subducting slabs are relatively old, slower imposed convergence tends to result in faster slab rollback such that the subduction rate does not change significantly. Thus, the effect of convergence rate on the slab surface temperature is relatively small (Supplementary Fig. 4).

## Discussion

Previous numerical studies that employ visco-plastic or visco-elasto-plastic rheology indicate that SSI is likely not mechanically plausible (e.g., refs. ^11,46,47^). In our study, SSI can occur when certain conditions are met, i.e., large age offset (>~80 Ma) and very young overriding plate (≤~20 Ma). The outcome of our study, however, depends on the rheological parameters for the lithosphere and the asthenosphere and the strength and the width of the weak zone that are implemented in our model. Given the uncertainties in these parameters, SSI cannot be excluded as a potential SI mode although SSI is likely less common than ISI in nature based on the specific conditions that it must satisfy.

Our modeling results indicate that the tip of the slab can become as hot as ~900 °C at 1 GPa during SSI (Fig. 2b). We refer to the range of the model-predicted PT-paths for the slab tip during SSI as the “high T” range (bounded by solid red lines in Fig. 2b). The high slab tip temperatures during SSI are attributed to the hot asthenosphere that upwells between two plates as the slab rolls back. This provides favorable thermal conditions for the formation of metamorphic soles. The slab tip during ISI can be almost as hot as that during SSI if the overriding plate is extremely young at ~1 Ma or less (beyond the region bounded by blue lines but below dotted blue line in Fig. 2b), for which the plate is at or in the vicinity of the spreading center. Therefore, metamorphic soles that are in the high T range likely formed during SSI unless the overriding plate is extremely young, in which case ISI is also possible.

To infer the slab surface PT condition during SI, we choose to use the metamorphic soles with the peak T conditions and not those with the peak P conditions because the highest temperature within a metamorphic sole is likely to be achieved along with the plate interface and also because the thermal response (i.e., temperature increase) could be delayed after reaching the peak P condition. The majority of metamorphic soles fall in the PT path range for the slab tip during ISI with an overriding plate of ~1–5 Ma in age (region bounded by solid blue lines in Fig. 2b). We refer to this range as the “intermediate T” range. The temperature along the slab surface decreases up-dip away from the slab tip, and this temperature range can also be produced during SSI, somewhere up-dip from the slab tip. If the age of the overriding plate can be identified to be older than 5 Ma, then the SI mode is likely SSI. However, if the plate is <5 Ma, the temperature alone cannot differentiate the two SI modes unless the location of their formation relative to the slab tip can be identified. Based on our modeling results, however, the relatively high thermal gradient (~20–40 °C/km) recorded by metamorphic soles can only be reproduced if they are at or near the slab tip and during the early stage of SI. For those that are in the lower temperature range (the “low T” range), the SI modes are even more difficult to differentiate based on the PT estimates from the metamorphic soles. Nonetheless, the majority of metamorphic soles fall in the intermediate to high temperatures ranges, and for them to have formed during ISI, the overriding plate must have been younger than ~5 Ma. Therefore, the age of the overriding plate holds the key to differentiating the two modes.

Studies on plate reconstruction and field observations indicate that the localities #4–21 were likely in intra-oceanic settings^7,30^ (blue stars in Fig. 2b). The SI at the Cuba locality (#4) occurred at a back-arc spreading center of a mature subduction zone or a fossil subduction zone^18,48^. Subduction at Oman (#13) is considered to have initiated along a mid-ocean ridge^34^. However, recent research indicates that Oman SI occurred at a fracture zone or a transform fault with a very young oceanic plate^15,18,49,50^. The petrological observations of the Saga ophiolite and the Lhasa metamorphic soles in Tibet (#14) indicate that the subduction also initiated at the back-arc or intra-arc spreading center^51,52^. Tasmania SI (#21) is also suggested near the back-arc spreading center^53^. For the localities in the Southwest Pacific areas (#17–20), both the overriding and subducting plates were young^7^, and in particular, it is proposed that SI occurred along a mid-ocean ridge or a back-arc spreading center for Palawan (#17)^35^ and New Caledonia (#20)^54^. In the SZI database of Crameri et al.^18^, the SI at Palawan (#17) is thought to be a polarity reversal event. The PT estimates for Palawan falls way beyond the PT ranges that our hottest cases of both SSI and ISI predict, indicating potentially anomalous processes or our model underpredicting the slab surface temperature during SI at a spreading center. At or near a spreading center, the negative buoyancy of either plate is not enough to drive SI spontaneously, and external forces are necessary to initiate new subduction. These localities (#4, 14, and 17–21) fall in the intermediate to high-temperature range and are consistent with our model prediction for ISI with a young overriding plate.

For localities that are associated with the subduction of the Neotethys (#7, 8, 9, 10, 12, and 13), previous studies indicate ISI as the SI mode^15,30,55^. Our modeling results indicate that they fall in the intermediate temperature range, for which a relatively young overriding plate is required for ISI. This is consistent with plate reconstruction studies that indicate relatively young overriding plates for these localities^7^ although the possibility of SSI cannot be ruled out from our modeling results.

For Newfoundland (#5), SI at a transform fault or fracture zone is proposed^56,57^. However, for this locality and Quebec (#6) and Egypt (#11), the plate ages are not well constrained, and the SI modes cannot be differentiated. The SI mode of the Nagaland locality in the low-T region (#15) cannot be differentiated based solely on the temperature conditions; the metamorphic soles may have formed either during SSI up-dip from the slab tip or during ISI, although a petrologic study suggests intra-oceanic ISI^58^. For the three localities along the western margin of North America (localities #1–3) and Andaman (#16), the overriding plate was continental and colder^7,59^. The PT conditions at these localities fall in the intermediate temperature range. Based on our modeling results, the cold overriding plate and the intermediate temperature range favor neither SSI nor ISI. However, some regional studies on Cascadia, California, and Andaman (#2, 3, 16) suggest ISI due to subduction polarity reversal^7,59^. The dynamics of subduction polarity reversal and its impact on the slab surface temperature are not included in our model and may contribute to anomalous conditions.

Besides metamorphic soles, magmatism, another crucial process in SI, may also help to differentiate SSI and ISI. Recent International Ocean Discovery Program (IODP) expeditions revealed detailed magmatic evolution during SI in IBM, where metamorphic soles have not been identified. Their results indicate that proto-forearc basalts, which are more generally referred to as forearc basalts (FABs), are the most abundant and oldest igneous rocks in the IBM forearc. These rocks formed by decompression melting of the depleted mantle with elevated mantle temperature during rapid forearc spreading at the beginning of SI^60^ and indicate the likeliness of SSI. Boninites that are also present in the IBM forearc were formed by flux melting of the highly depleted mantle residue at high-temperature and low pressure^61,62^. Both FABs and boninites are common components of SSZ ophiolites^63^ elsewhere. What is considered another indicator of SSI for IBM is the presence of adakites^64^, which form from partial melting of the subducting oceanic crust. Adakites are not a usual component in SSZ ophiolites, and their formation requires that the subducting slab surface must be anomalously hot, which is difficult to explain with ISI, given the old age of the subducting plate that is inferred for IBM. The magmatic evolution in IBM, therefore, indicates SSI. However, the presence of Mesozoic aged materials near the present-day trench has been interpreted to indicate an old overriding plate (~100 Ma) at the time of SI^23,65^. If this interpretation is correct, SSI would have been difficult due to the lack of thermal contrast between the two plates, indicating ISI. However, with ISI, proto-forearc spreading and the formation of adakites are difficult to explain. These contrasting observables require further investigation. In some subduction zones, such as Puysegur and New Caledonia, the plate convergence and the lack of forearc spreading indicate ISI, and the occurrence of adakites is attributed to the young subducting plate, which provides the hot condition required for partial melting of the subducting crust^66–68^.

A recent numerical study indicates that FABs do not form during ISI and that the presence of FABs indicates SSI^47^. In their SI model, initial vertical pull and/or horizontal compression is imposed on one of the two plates as an internal force boundary condition to induce subduction initiation. The vertical pull results in the sinking of the subducting slab into the asthenosphere and rapid forearc spreading, quite similar to our SSI models, promoting the formation of FABs, boninites and adakites. In contrast, the application of the horizontal compression leads to ISI without FABs in most of their models. In our ISI models, however, forearc spreading occurs when the subduction rate is relatively low (e.g., 1 cm/yr; Supplementary Fig. 2g–i), and therefore the formation of FABs is not limited to SSI. In this situation, the formation of SSZ ophiolites occurs after the formation of metamorphic soles, consistent with the previous geochronological observations^15^. Thus, it is the timing of the SSZ ophiolite formation relative to the metamorphic sole formation, not the presence or the absence of FABs, that can help differentiate the two SI modes.

Shear heating has been invoked to reconcile the inconsistency in the PT conditions between the thermal models and metamorphic rocks. Based on recent analytical and numerical modeling work by van Keken et al.^69,70^, however, the temperature raised by shear heating is less than 50^o^C for a commonly assumed range of effective frictional coefficient (0.025–0.05; see refs. ^71–73^). Thus, the amount of temperature increase is relatively small and does not influence the conclusion of this paper. The tectonic pressure that is recorded by metamorphic soles can impact the interpretation of the metamorphic soles^74^. Li et al.^75^ pointed out that the tectonic overpressure can give rise to pressure deviation on the order of 0.3 GPa (10–20% of lithostatic pressure) for the HP-UHP rocks. If the metamorphic soles were undergoing tectonic overpressure, removal of this effect will result in an increase in the thermal gradient, and some of the highest thermal gradients (e.g., localities #19, #20, and #21) can be even more challenging to explain with our models, even with an extremely young overriding plate in either SI mode. Further detailed geological observations and numerical modeling is needed to resolve this issue.

Our modeling results indicate that SSI can lead to relatively high slab surface temperatures for wider ranges of subduction parameter values compared to ISI, indicating that SSI would be more favorable for the formation of HT-LP metamorphic soles. However, the above synthesis of our modeling results with the geological observations indicates that the majority of HT-LP metamorphic soles likely formed during ISI that involved a very young (<5 Ma) overriding plate, and in many cases, the subducting plate was also very young, likely indicating SI along spreading centers. These observations point to the likeliness of future metamorphic sole formation during SI in young oceanic basins (e.g., in Southwest Pacific). However, there could be many paleo and active subduction zones, for which metamorphic soles were not preserved, and it is unclear whether the preserved metamorphic soles indicate the PT conditions that are representative of all SI or biased towards certain conditions (e.g., high-temperature conditions). Thus, our conclusion that the ISI is more common than SSI applies to the population of paleo- and active subduction zones with preserved metamorphic soles.

## Methods

### Governing equations

We use the geodynamic code I2VIS to solve the continuity and momentum (Stokes) equations for incompressible flow and the energy equation, using the finite difference and marker-in-cell methods with irregular staggered Eulerian grids:

1 $$\nabla \cdot {{{{{\bf{v}}}}}}=0$$

2 $$\nabla \cdot {{{{{{\boldsymbol{\sigma }}}}}}}^{{{\prime}}}-\nabla P+\rho {{{{{\bf{g}}}}}}=0$$

3 $$\rho {C}_{{{{{{\mathrm{P}}}}}}}\frac{{{{{{{\mathrm{D}}}}}}T}}{{{{{{{\mathrm{D}}}}}}t}}=\nabla \cdot \left(k\nabla T\right)+{H}_{{{{{{\mathrm{r}}}}}}}+{H}_{{{{{{\mathrm{a}}}}}}}+{H}_{{{{{{\mathrm{s}}}}}}}$$

where ${{{{{{\boldsymbol{\sigma }}}}}}}^{{{{\prime} }}}$ is the deviatoric stress tensor, **v** is the velocity vector, **g** is gravitational acceleration, *P* is dynamic pressure, $\rho$ is density, *C*_p_ is the heat capacity, *T* is temperature, D*T*/D*t* is the substantive (Lagrangian) time derivative of temperature, *H*_r_ is radioactive heat production, *H*_a_ is adiabatic heating, and *H*_s_ is shear heating. The deviatoric stress tensor is defined as

4 $${{{{{{\boldsymbol{\sigma }}}}}}}^{{{{\prime} }}}=2{\eta }_{{{{{{\rm{eff}}}}}}}\dot{{{{{{\boldsymbol{\varepsilon }}}}}}}$$

5 $${\dot{\varepsilon }}_{{ij}}=\frac{1}{2}\left(\frac{\partial {v}_{i}}{\partial {x}_{j}}+\frac{\partial {v}_{j}}{\partial {x}_{i}}\right)$$

where $\dot{{{{{{\boldsymbol{\varepsilon }}}}}}}$ is the deviatoric strain rate tensor, ${\dot{\varepsilon }}_{{ij}}$ is a component of the deviatoric strain rate tensor, which is calculated from components of the velocity vector, *v*_*i*_ and *v*_*j*_, ${\eta }_{{{{{{\rm{eff}}}}}}}$ is the effective viscosity, which is defined below. The density in the momentum equation (Eq. 2) depends on the lithology, pressure, and temperature:

6 $${\rho }_{P,T}={\rho }_{0}\left[1-\alpha (T-{T}_{0})\right]\left[1+\beta (P-{P}_{0})\right]$$

where $\alpha =3\times {10}^{-5}\,{{{{{{\rm{K}}}}}}}^{-1}$ and $\beta =1\times {10}^{-5}\,{{{{{{\rm{MPa}}}}}}}^{-1}$ are the thermal expansion and compressibility coefficients, respectively, and the parameters with a subscript “0” indicates the density reference value for a given lithology. Detailed numerical approaches are described in previous studies^76,77^.

### Numerical model setup

Two initial model setups were explored that correspond to the two SI modes (Fig. 3 and Supplementary Fig. 1). The first model setup is for SSI, for which SI is driven only by the negative buoyancy. A similar model setup has been used to investigate the SSI^46,75^. The geometry of the initial 2-D model is 2000-km wide and 680-km deep with varying resolution from 1 km $\times$ 1 km near the weak zone to 10 km $\times$ 10 km near the boundaries (Supplementary Fig. 1). Each grid cell has 12 randomly distributed markers that are used to trace rock composition, material properties, and temperature. A similar model setup using I2VIS has been performed and tested in previous research^78,79^. For the initial condition, a weak zone is placed between a young and an old oceanic plate, representing a transform fault/fracture zone. The oceanic crust consists of a 3-km-thick basalt and 5-km-thick gabbro layers, and the thickness of the oceanic lithosphere is thermally defined. The initial temperature field for the oceanic lithosphere is calculated by using the half-space cooling model with 0 °C at the surface and 1350 °C for the mantle potential temperature. The adiabatic temperature gradient of 0.5 °C/km is applied to the asthenosphere. Free slip condition is applied to all of the boundaries. An internal free surface boundary is implemented by applying a 10-km-thick “sticky air” at the surface^80–82^. The initial geotherms for these plates are calculated for 20- and 100-Myr-old plates. The weak zone between the plates is 30-km wide and 50-km deep. The rheological parameters and material properties are listed in Supplementary Tables 2 and 3.

### Rheology

A visco-plastic rheology is applied for the entire model domain with different rheological parameters for compositionally distinct layers^83,84^ (Fig. 3). The model does not include elasticity, and quantitative analyses of the bending force on the subducting slab during SI may need to be addressed elsewhere. For each rock type, the “ductile” viscosity and the “plastic” viscosity are calculated separately; the latter controls the effect of brittle deformation in the model. For the ductile viscosity of the oceanic crust, the flow law for dislocation creep in Ranalli^84^ is used:

7 $${\eta }_{{{{{{{\mathrm{ductile}}}}}}}}=\frac{1}{2}{\left({A}_{{{{{{\mathrm{R}}}}}}}\right)}^{-\frac{1}{n}}{({\dot{\varepsilon }}_{{II}})}^{\frac{1-n}{n}}{\exp }\left(\frac{E+{PV}}{{nRT}}\right)$$

where ${\eta }_{{{{{{{\mathrm{ductile}}}}}}}}$ is the ductile viscosity that accounts only for a dislocation-creep rheology, ${\dot{\varepsilon }}_{{II}}\,$ is the second invariant of the strain rate tensor, *R* is the gas constant, ${A}_{{{{{{\mathrm{R}}}}}}}\,$ is a pre-exponential factor, *E* is the activation energy, *V* is activation volume, and *n* is the creep/stress exponent. For the ductile viscosity of the mantle, the composite rheology is used to account for the effect of both diffusion creep and dislocation creep^83^. The flow law to calculate the dislocation-creep and diffusion-creep viscosity is given by

8 $${\eta }_{{{{{{\rm{diffusion}}}}}}/{{{{{\rm{dislocation}}}}}}}=\frac{1}{2}{({A}_{K})}^{-\frac{1}{n}}\mu {\left(\frac{d}{b}\right)}^{\frac{m}{n}}{\left({\dot{\varepsilon }}_{{II}}\right)}^{\frac{1-n}{n}}{\exp }\left(\frac{E+{PV}}{{nRT}}\right)$$

where *A*_K_ is a pre-exponential factor, $\mu$ is shear modulus (80 GPa), *d* is mineral grain size (1 mm), *b* is the length of Burgers vector (0.5 mm), *m* is the grain size exponent. The effective ductile viscosity of a given lithology is described by

9 $${\eta }_{{{{{{{\mathrm{ductile}}}}}}}}={\left(\frac{1}{{\eta }_{{{{{{{\mathrm{diffusion}}}}}}}}}+\frac{1}{{\eta }_{{{{{{{\mathrm{dislocation}}}}}}}}}\right)}^{-1}$$

For the plastic viscosity calculation, the extended Drucker–Prager yield criterion^84^ is implemented:

10 $${\sigma }_{{{{{{\rm{yield}}}}}}}={C}_{0}+{\sigma }_{N}{{\sin }}\left({\varphi }_{{{{{{\rm{eff}}}}}}}\right)$$

11 $${\eta }_{{{{{{\rm{plastic}}}}}}}=\frac{{\sigma }_{{{{{{\rm{yield}}}}}}}}{2{\dot{\varepsilon }}_{{{{{{\rm{II}}}}}}}}$$

where ${\sigma }_{{{{{{\rm{yield}}}}}}}$ is the yield stress, ${\sigma }_{N}$ is the normal stress on the fault, which is approximated by the lithostatic pressure, ${\varphi }_{{{{{{\rm{eff}}}}}}}\,$ is the effective internal friction angle, and *C*_0_ is cohesion. The effective viscosity of a given lithology is defined by the minimum value of the ductile and plastic viscosities,

12 $${\eta }_{{{{{{\rm{eff}}}}}}}={\min }\left({\eta }_{{{{{{\rm{ductile}}}}}}},{\eta }_{{{{{{\rm{plastic}}}}}}}\right).$$

Rheological parameter values used in the models are summarized in Supplementary Table 2, and other material parameter values are summarized in Supplementary Table 3.

In the ISI case, we implemented an internal velocity of 3 cm/yr on the old plate. In both models, 12 tracers are placed randomly within each cell. We identify a tracer that is closest to each of the nodes that define the base of the 3-km-thick upper oceanic crust and use these tracers to approximate the temperature condition along with the subduction interface. We avoid using tracers that are closer to the top of the upper oceanic crust because they can become detached from the subducting slab due to the particular set of rheological parameter values that are used in the models.

## Supplementary information

Supplementary file

## Data Availability

Data used in this study are from the published literature and can be founded in the corresponding references. All numerical model output is available from the corresponding author upon request.

## Code availability

Requests for the numerical code I2VIS should be sent to the main developer (taras.gerya@erdw.ethz.ch).
