# Supplementary material for: Differentiating induced versus spontaneous subduction initiation using thermomechanical models and metamorphic soles
Source: Nat Commun. 2021 Jul 30;12:4632. doi: 10.1038/s41467-021-24896-x (PMC8324860; doi:10.1038/s41467-021-24896-x)
Supplement: Supplementary file 1 — Supplementary file [file 41467_2021_24896_MOESM1_ESM.pdf]

Supplementary Material

Differentiating Induced versus Spontaneous Subduction Initiation Using Thermomechanical Models and Metamorphic Soles

Xin Zhou[1,*] and Ikuko Wada[1]

[1]Department of Earth and Environmental Sciences, University of Minnesota

116 Church Street SE, Minneapolis, MN 55455, USA

e-mail: zhou1482@umn.edu

Supplementary Table 1. Compiled data of metamorphic soles (modified from ref.[1])

Locality number	Locality name	Possible Tectonic setting before SI	Proposed SI mode	P (GPa)	T(^{o}C)	Age (Ma)	References[*]
1	Books Range	backarc center near passive margin	unknown	0.5	600-700	169	refs.[2,3]
2	Cascadia	passive margin	unknown	1.0	~760	> 167	ref.[4]
3	California	transform fault, passive margin	SSI	0.95	645	180	refs.[5–7]
4	Cuba	spreading ridge, fossil subduction zone	ISI	0.85-0.87	650-665	90-70,49	refs.[8,9]
5	Newfound	transform fault	SSI	0.9	800	485	refs.[10–13]
6	Quebec	oceanic basin	unknown	0.95	850	476	ref.[14]
7	Dinarides	spreading ridge, seamount boundary	ISI	1.1-1.2	745-830	170	refs.[15–20]
8	Turkey	oceanic basin	unknown	1.05	800	93	refs.[21–31]
9	Caucasus	oceanic basin	unknown	0.65	630	91-94	refs.[32–37]
10	Cyclades	oceanic basin	unknown	0.85	750	162	refs.[38–40]
11	Egypt	oceanic basin	unknown	0.60	720	630-590	refs.[41,42]
12	Syria	unknown	unknown	unknown	>=600	93	ref.[43]
13	Oman	transform fault, spreading ridge	ISI	1.16	840	96-92	refs.[44–47]
14	Tibet	spreading ridge	ISI	1.2	850	130	refs.[48–53]
15	Nagaland	oceanic basin	ISI	1.38	625	116	ref.[54]
16	Andaman	spreading ridge near passive margin	ISI	0.95	675	106	ref.[55]
17	Palawan	spreading ridge	ISI	0.90	760	30	refs.[56,57]
18	Sulawesi	spreading ridge	ISI	unknown	700	60	ref.[58]
19	Papua	unknown	unknown	0.4	900	58	ref.[59]
20	New Caledonia	spreading ridge	ISI	0.6	800	55	ref.[60]
21	Tasmania	spreading ridge	ISI	0.8	875	513	ref.[61]

* Also see the references in ref.[1]

Supplementary Table 2. Rheological parameters for the viscous flow laws used in the numerical experiments

**Flow Laws**	**E** (kJ mol^{-1})	**V** (J MPa^{-1}mol^{-1})	**n**	$\mathbf{A_R}$ (MPa^{-n} s^{-1})	m	$\mathbf{A_k}$ (s^{-1})
Air/water	0	0	1	1.0×10^{-22}	N/A	N/A
Wet quartzite	154	0	2.3	3.2×10^{-4}	N/A	N/A
Plagioclase An75	238	0	3.2	3.3×10^{-4}	N/A	N/A
Dry olivine/dislocation	540	15	3.5	N/A	0	3.5×10^{22}
Dry olivine/diffusion	300	4	1	N/A	2.5	8.7×10^{15}
Wet olivine/dislocation	430	10	3	N/A	0	2.0×10^{18}
Wet olivine/diffusion	240	5	1	N/A	2.5	5.3×10^{15}

Supplementary Table 3. Material properties used in the numerical experiments*

Material	ρ_0 (kg m^{-3})	C_p (J kg^{-1}K^{-1})	k (Wm^{-1}K^{-1})	H_r (uWm^{-3})	Plastic C_0 (MPa)	Plastic sin (φ_{eff})
Sticky air	1	3.33×10^6	200	0	0	0
Water	1000	3330	200	0	0	0
Upper oceanic crust	3000	1000	K_1**	0.25	3	0
Lower oceanic crust	3000	1000	K_1**	0.25	3	0.3
Weak zone	3300	1000	K_2**	0.022	3	0
Mantle	3300	1000	K_2**	0.022	3	0.6

* The values are obtained from refs.[62–66].

** $K_1 = [1.18 + 474/(T_K + 77)] - \exp(0.00004P_{MPa})$, $K_2 = [0.73 + 1293/(T_K + 77)] - \exp(0.00004P_{MPa})$.

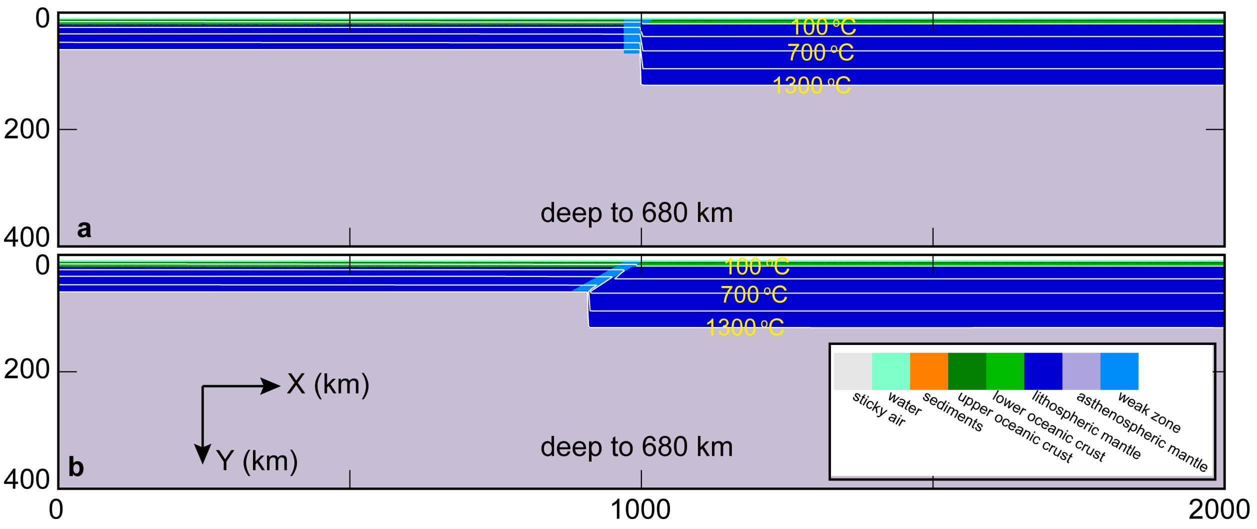

Supplementary Figure 1. Initial model setup. **a** spontaneous subduction initiation (SSI) and **b** induced subduction initiation (ISI).

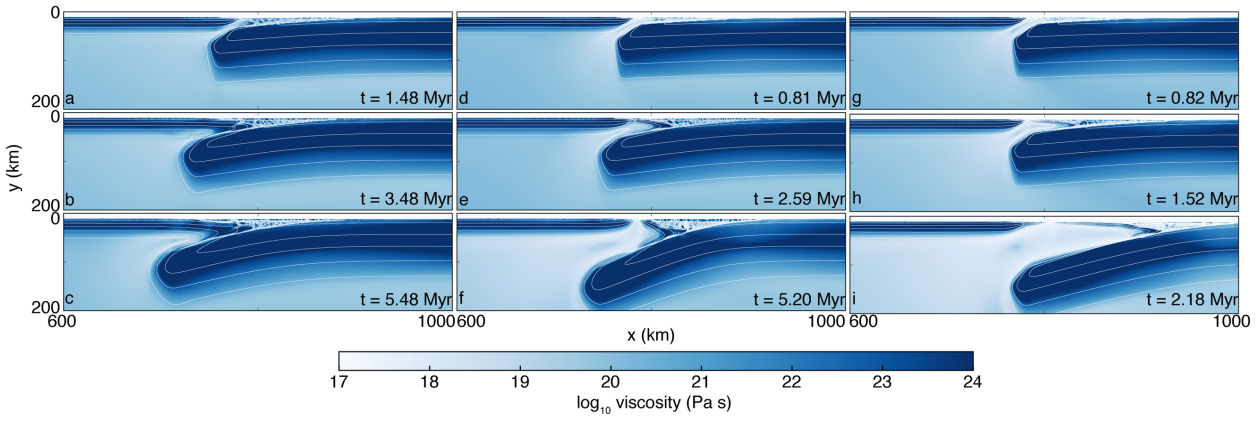

Supplementary Figure 2. Effects of the convergence rate on proto-forearc spreading. The models are for induced subduction initiation (ISI) with a 100-Ma subducting slab age, a 5-Ma overriding plate, and three different convergence rates: **a-c** 3 cm/yr, without proto-forearc spreading, **d-f** 2 cm/yr, with slow proto-forearc spreading, and **g-i** 1 cm/yr, with extensive proto-forearc spreading.

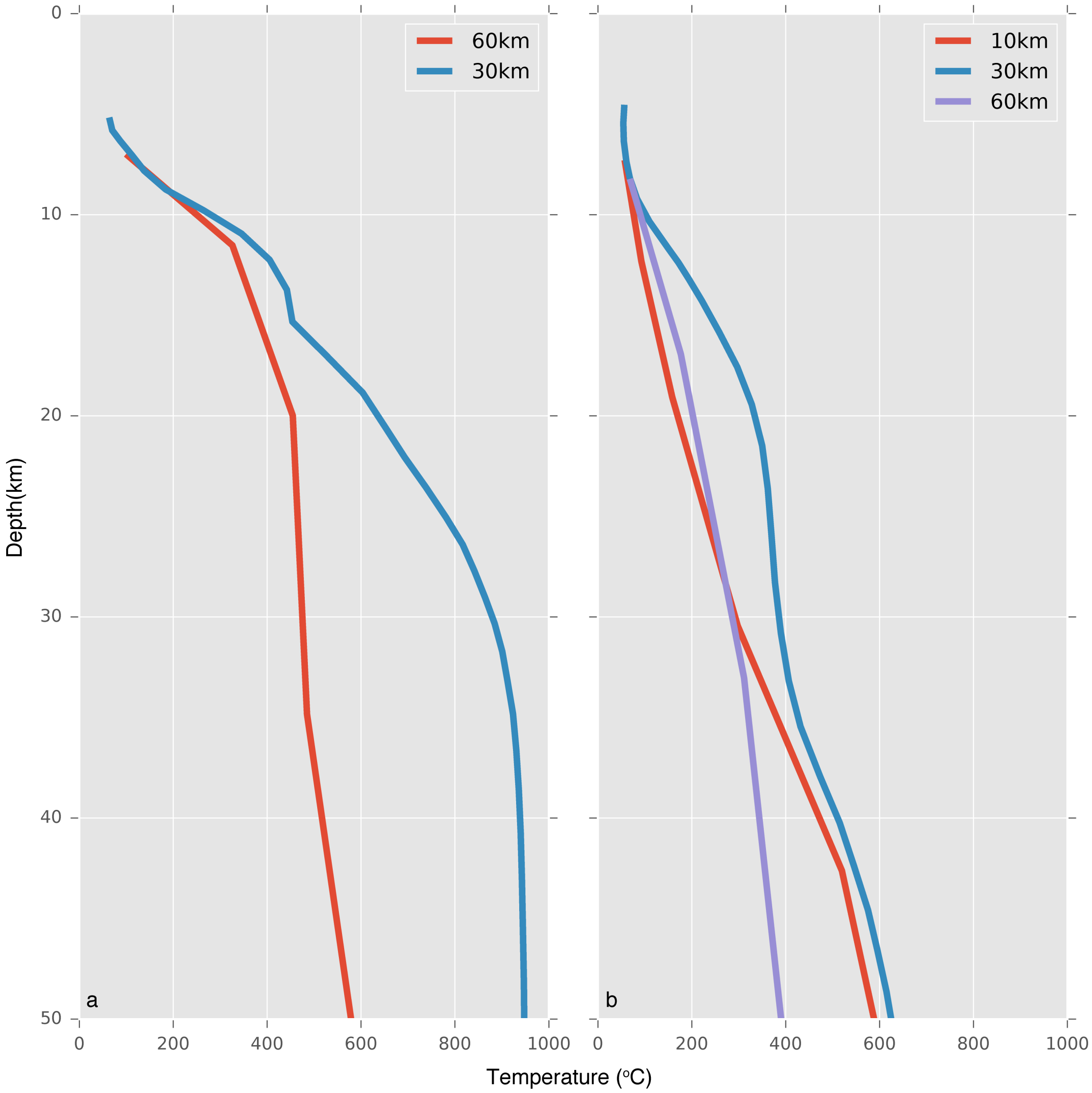

Supplementary Figure 3. Effect of the weak zone on slab surface temperature. Variation in the P-T path of a tracer along the slab surface at 10 km from the slab tip for different widths of the weak zone for **a.** spontaneous subduction initiation (SSI) and **b.** induced subduction initiation (ISI). All model parameters except the weak zone width are identical to those in the reference SSI and ISI models, respectively.

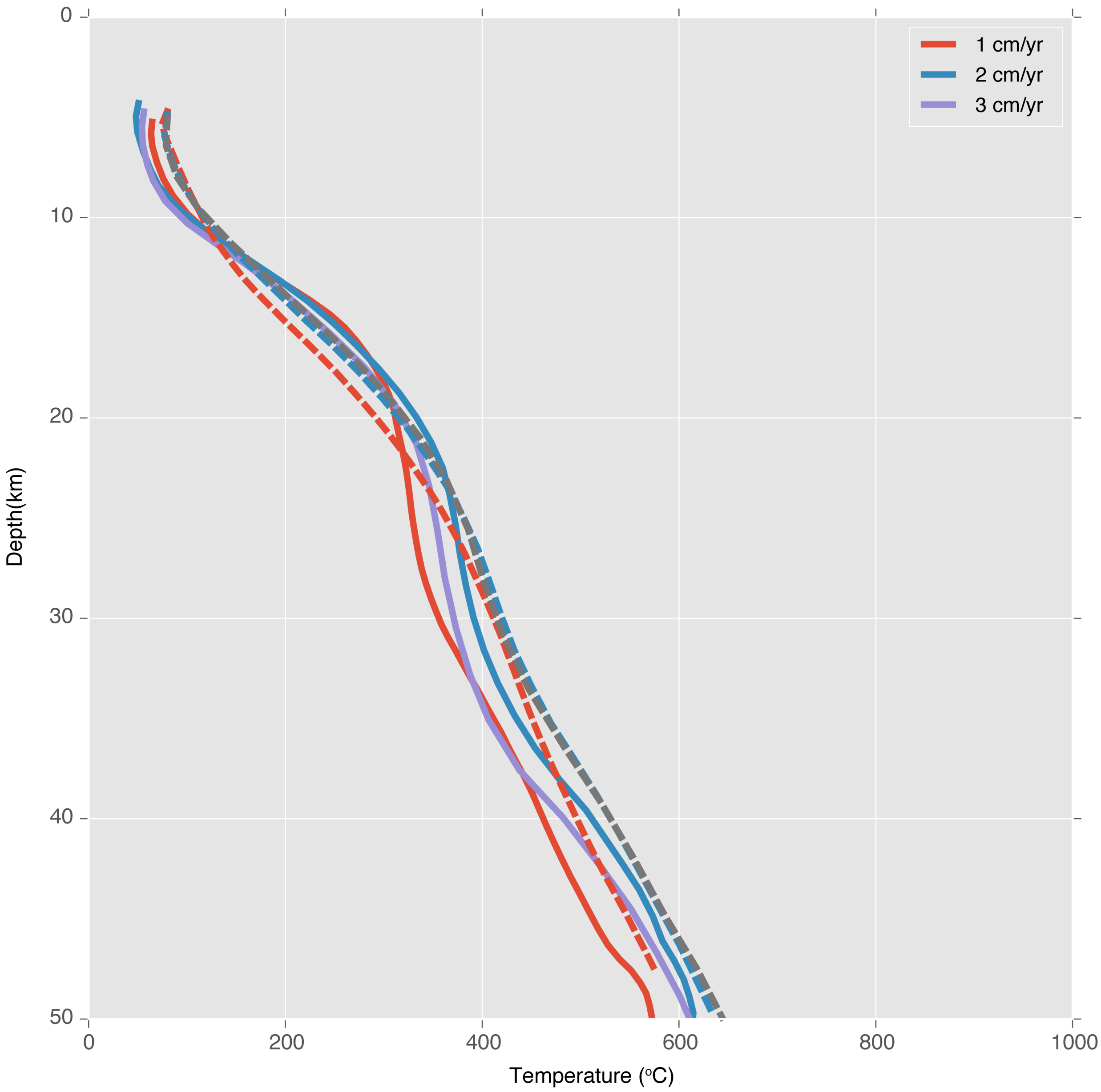

Supplementary Figure 4. Effect of the convergence rate on slab surface temperature. Variation in the P-T path of a tracer along the slab surface at 10 km from the slab tip for different imposed convergence rates for ISI with the subducting slab age of 100 Ma (solid lines) and 50 Ma (dashed lines). All model parameters except the convergence rate and the subducting slab age are identical to those in the reference ISI model.

Supplementary References

1. Agard, P. *et al.* Plate interface rheological switches during subduction infancy: Control on slab penetration and metamorphic sole formation. *Earth Planet. Sci. Lett.* **451**, 208–220 (2016).
2. Harris, R. A. Origin and tectonic evolution of the metamorphic sole beneath the Brooks Range ophiolite, Alaska. *Spec. Pap. Geol. Soc. Am.* **324**, 293–312 (1998).
3. Harris, R. Tectonic evolution of the Brooks Range ophiolite, northern Alaska. *Tectonophysics* **392**, 143–163 (2004).
4. Cordova, J. L. *et al.* Subduction initiation and early evolution of the Easton metamorphic suite, northwest Cascades, Washington. *Lithosphere* **11**, 44–58 (2019).
5. Wakabayashi, J. Counterclockwise PTt paths from amphibolites, Franciscan Complex, California: Relics from the early stages of subduction zone metamorphism. *J. Geol.* **98**, 657–680 (1990).
6. Rutte, D., Garber, J., Kylander-Clark, A. & Renne, P. R. An Exhumation Pulse From the Nascent Franciscan Subduction Zone (California, USA). *Tectonics* **39**, (2020).
7. Shervais, J. W. & Choi, S. H. Subduction initiation along transform faults: The proto-franciscan subduction zone. *Lithosphere* **4**, 484–496 (2012).
8. Lázaro, C. *et al.* First description of a metamorphic sole related to ophiolite obduction in the northern Caribbean: Geochemistry and petrology of the Güira de Jauco Amphibolite complex (eastern Cuba) and tectonic implications. *Lithos* **179**, 193–210 (2013).
9. Crameri, F. *et al.* A transdisciplinary and community-driven database to unravel subduction zone initiation. *Nat. Commun.* **11**, 1–14 (2020).
10. Cawood, P. A. & Suhr, G. Generation and obduction of ophiolites: constraints from the Bay of Islands Complex, western Newfoundland. *Tectonics* **11**, 884–897 (1992).
11. Dewey, J. F. & Casey, J. F. The sole of an ophiolite: The Ordovician Bay of Islands Complex, Newfoundland. *J. Geol. Soc. London.* **170**, 715–722 (2013).
12. Fergusson, C. L. & Cawood, P. A. Structural history of the metamorphic sole of the Bay of Islands

Complex, western Newfoundland. *Can. J. Earth Sci.* **32**, 533–544 (1995).

13. SUHR, G. & Cawood, P. A. Structural history of ophiolite obduction, Bay of Islands, Newfoundland. *Geol. Soc. Am. Bull.* **105**, 399–410 (1993).

14. Dubacq, B., Soret, M., Jewison, E. & Agard, P. Early subduction dynamics recorded by the metamorphic sole of the Mt. Albert ophiolitic complex (Gaspé Quebec). *Lithos* **334–335**, 161–179 (2019).

15. Balen, D. & Massonne, H. J. A PT path for amphibolites from the metamorphic sole of the Dinaride ophiolite zone in Bosnia. in *AGU Fall Meeting Abstracts* **2016**, V33D--3145 (2016).

16. Šegvić, B. *et al.* Sm-Nd geochronology and petrologic investigation of a sub,ophiolite metamorphic sole from the dinarides (Krivaja-konjuh ophiolite complex, Bosnia and Herzegovina). *Geol. Croat.* **73**, 1–11 (2020).

17. Šegvić, B. *et al.* Petrogenesis of high-grade metamorphic soles from the central dinaric ophiolite belt and their significance for the neotethyan evolution in the dinarides. *Ofioliti* **44**, 1–30 (2019).

18. Pamić, J., Tomljenović, B. & Balen, D. Geodynamic and petrogenetic evolution of Alpine ophiolites from the central and NW Dinarides: An overview. *Lithos* **65**, 113–142 (2002).

19. Gaggero, L., Marroni, M., Pandolfi, L. & Buzzi, L. Modeling the oceanic lithosphere obduction : Constraints from the metamorphic sole of Mirdita ophiolites (northern Albania). *Ofioliti* **34**, 17–42 (2009).

20. Borojević Sostarić, S. *et al.* The origin and age of the metamorphic sole from the Rogozna Mts., Western Vardar Belt: New evidence for the one-ocean model for the Balkan ophiolites. *Lithos* **192–195**, 39–55 (2014).

21. Polat, A., Casey, J. F. & Kerrich, R. Geochemical characteristics of accreted material beneath the Pozanti-Karsanti ophiolite, Turkey: Intra-oceanic detachment, assembly and obduction. *Tectonophysics* **263**, 249–276 (1996).

22. Plunder, A. *et al.* Metamorphic sole formation, emplacement and blueschist facies overprint: early subduction dynamics witnessed by western Turkey ophiolites. *Terra Nov.* **28**, 329–339 (2016).

23. Çelik, Ö. F. *et al.* Early-Middle Jurassic intra-oceanic subduction in the Izmir-Ankara-Erzincan Ocean, Northern Turkey. *Tectonophysics* **509**, 120–134 (2011).

24. Plunder, A., Agard, P., Chopin, C. & Okay, A. I. Geodynamics of the Tavşanli{dotless} zone, western Turkey: Insights into subduction/obduction processes. *Tectonophysics* **608**, 884–903 (2013).

25. Parlak, O. *et al.* Rapid cooling history of a Neotethyan ophiolite: Evidence for contemporaneous subduction initiation and metamorphic sole formation. *Bull. Geol. Soc. Am.* **131**, 2011–2038 (2019).

26. Parlak, O., Yilmaz, H. & Boztuğ, D. Origin and tectonic significance of the metamorphic sole and isolated dykes of the Divriği ophiolite (Sivas, Turkey): Evidence for slab break-off prior to ophiolite emplacement. *Turkish J. Earth Sci.* **15**, 25–45 (2006).

27. Çelik, Ö. F. Metamorphic sole rocks and their mafic dykes in the eastern Tauride belt ophiolites (southern Turkey): Implications for OIB-type magma generation following slab break-off. *Geol. Mag.* **144**, 849–866 (2007).

28. Çelik, Ö. F. & Delaloye, M. F. Origin of metamorphic soles and their post-kinematic mafic dyke swarms in the Antalya and Lycian ophiolites, SW Turkey. *Geol. J.* **38**, 235–256 (2003).

29. Daşçi, H. T., Parlak, O., Nurlu, N. & Billor, Z. Geochemical characteristics and age of metamorphic sole rocks within a Neotethyan ophiolitic mélange from Konya region (central southern Turkey). *Geodin. Acta* **27**, 223–243 (2015).

30. Elitok, Ö. & Drüppel, K. Geochemistry and tectonic significance of metamorphic sole rocks beneath the Beyşehir-Hoyran ophiolite (SW-Turkey). *Lithos* **100**, 322–353 (2008).

31. Dilek, Y. & Whitney, D. L. Counterclockwise P-T-t trajectory from the metamorphic sole of a Neo-Tethyan ophiolite (Turkey). *Tectonophysics* **280**, 295–310 (1997).

32. Haessig, M., Rolland, Y., Sosson, M. & Avagyan, A. Lithological nature of the subduction channel: Insights from the Karabakh suture zone (Lesser Caucasus) and general comparisons. *J. Geodyn.* **96**, 19–34 (2016).

33. Rolland, Y. *et al.* The East Anatolia–Lesser Caucasus ophiolite: An exceptional case of large-scale obduction, synthesis of data and numerical modelling. *Geosci. Front.* **11**, 83–108 (2020).

34. Hässig, M. *et al.* Linking the NE Anatolian and Lesser Caucasus ophiolites: Evidence for large-scale obduction of oceanic crust and implications for the formation of the Lesser Caucasus-Pontides Arc. *Geodin. Acta* **26**, 311–330 (2013).

35. Hässig, M., Rolland, Y. & Sosson, M. From seafloor spreading to obduction: Jurassic-Cretaceous evolution of the northern branch of the Neotethys in the Northeastern Anatolian and Lesser Caucasus regions. *Geol. Soc. Spec. Publ.* **428**, 41–60 (2017).

36. Hässig, M. *et al.* New structural and petrological data on the Amasia ophiolites (NW Sevan-Akera suture zone, Lesser Caucasus): Insights for a large-scale obduction in Armenia and NE Turkey. *Tectonophysics* **588**, 135–153 (2013).

37. Rolland, Y. *et al.* A review of the plate convergence history of the East Anatolia-Transcaucasus region during the Variscan: Insights from the Georgian basement and its connection to the Eastern Pontides. *J. Geodyn.* **96**, 131–145 (2016).

38. Katzir, Y., Matthews, A., Garfunkel, Z., Schliestedt, M. & Avigad, D. The tectono-metamorphic evolution of a dismembered ophiolite (Tinos, Cyclades, Greece). *Geol. Mag.* **133**, 237–254 (1996).

39. Lamont, T. *et al.* The Cycladic subduction zone from birth to death: Insights into the subduction cooling rate conundrum. in *EGU General Assembly Conference Abstracts* 21985 (2020).

40. Gartzos, E., Dietrich, V. J., Migiros, G., Serelis, K. & Lymperopoulou, T. The origin of amphibolites from metamorphic soles beneath the ultramafic ophiolites in Evia and Lesvos (Greece) and their geotectonic implication. *Lithos* **108**, 224–242 (2009).

41. Farahat, E. S. Geotectonic significance of Neoproterozoic amphibolites from the Central Eastern Desert of Egypt: A possible dismembered sub-ophiolitic metamorphic sole. *Lithos* **125**, 781–794 (2011).

42. Abd El-Naby, H., Frisch, W. & Hegner, E. Evolution of the Pan-African Wadi Haimur metamorphic sole, Eastern Desert, Egypt. *J. Metamorph. Geol.* **18**, 639–651 (2000).

43. Al-Riyami, K., Robertson, A., Dixon, J. & Xenophontos, C. Origin and emplacement of the Late Cretaceous Baer-Bassit ophiolite and its metamorphic sole in NW Syria. *Lithos* **65**, 225–260 (2002).

44. Soret, M., Agard, P., Dubacq, B., Plunder, A. & Yamato, P. Petrological evidence for stepwise accretion of metamorphic soles during subduction infancy (Semail ophiolite, Oman and UAE). *J. Metamorph. Geol.* **35**, 1051–1080 (2017).

45. Goscombe, B., Foster, D. A., Gray, D., Kelsey, D. & Wade, B. Metamorphic response within different subduction–obduction settings preserved on the NE Arabian margin. *Gondwana Res.* **83**, 298–371 (2020).

46. Soret, M. *et al.* Deformation mechanisms in mafic amphibolites and granulites: record from the Semail metamorphic sole during subduction infancy. *Solid Earth Discuss.* 1–36 (2019). doi:10.5194/se-2019-28

47. Guilmette, C. *et al.* Forced subduction initiation recorded in the sole and crust of the Semail Ophiolite of Oman. *Nat. Geosci.* **11**, 688–695 (2018).

48. Singh, A. K., Chung, S. L., Bikramaditya, R. K. & Lee, H. Y. New u–Pb zircon ages of plagiogranites from the Nagaland–Manipur Ophiolites, Indo-Myanmar Orogenic Belt, NE India. *J. Geol. Soc. London.* **174**, 170–179 (2017).

49. Guilmette, C. *et al.* Discovery of a dismembered metamorphic sole in the Saga ophiolitic mélange, South Tibet: Assessing an Early Cretaceous disruption of the Neo-Tethyan supra-subduction zone and consequences on basin closing. *Gondwana Res.* **22**, 398–414 (2012).

50. Guilmette, C., Hébert, R., Wang, C. & Villeneuve, M. Geochemistry and geochronology of the metamorphic sole underlying the Xigaze Ophiolite, Yarlung Zangbo Suture Zone, South Tibet. *Lithos* **112**, 149–162 (2009).

51. Guilmette, C., Hébert, R., Dupuis, C., Wang, C. & Li, Z. Metamorphic history and geodynamic significance of high-grade metabasites from the ophiolitic mélange beneath the Yarlung Zangbo ophiolites, Xigaze area, Tibet. *J. Asian Earth Sci.* **32**, 423–437 (2008).

52. Zhang, C. *et al.* Subduction re-initiation at dying ridge of Neo-Tethys: Insights from mafic and metamafic rocks in Lhaze ophiolitic mélange, Yarlung-Tsangbo Suture Zone. *Earth Planet. Sci. Lett.* **523**, 115707 (2019).

53. Guilmette, C. *et al.* Discovery of a dismembered metamorphic sole in the Saga ophiolitic mélange, South Tibet: Assessing an Early Cretaceous disruption of the Neo-Tethyan supra-subduction zone and consequences on basin closing. *Gondwana Res.* **22**, 398–414 (2012).

54. Bhowmik, S. K. & Ao, A. Subduction initiation in the Neo-Tethys: Constraints from counterclockwise P-T paths in amphibolite rocks of the Nagaland Ophiolite Complex, India. *J. Metamorph. Geol.* **34**, 17–44 (2016).

55. Plunder, A. *et al.* History of Subduction Polarity Reversal During Arc-Continent Collision: Constraints From the Andaman Ophiolite and its Metamorphic Sole. *Tectonics* **39**, 1–24 (2020).

56. Encarnación, J. P., Essene, E. J., Mukasa, S. B. & Hall, C. H. High-pressure and -temperature subophiolitic kyanite - garnet amphibolites generated during initiation of mid-tertiary subduction, Palawan, Philippines. *J. Petrol.* **36**, 1481–1503 (1995).

57. Keenan, T. E. *et al.* Rapid conversion of an oceanic spreading center to a subduction zone inferred from high-precision geochronology. *Proc. Natl. Acad. Sci. U. S. A.* **113**, E7359–E7366 (2016).

58. Parkinson, C. D. The origin and significance of metamorphosed tectonic blocks in mélanges: Evidence from Sulawesi, Indonesia. *Terra Nov.* **8**, 312–323 (1996).

59. Lus, W. Y., McDougall, I. & Davies, H. L. Age of the metamorphic sole of the Papuan Ultramafic Belt ophiolite, Papua New Guinea. *Tectonophysics* **392**, 85–101 (2004).

60. Cluzel, D., Jourdan, F., Meffre, S., Maurizot, P. & Lesimple, S. The metamorphic sole of New Caledonia ophiolite: 40Ar/ 39Ar, U-Pb, and geochemical evidence for subduction inception at a spreading ridge. *Tectonics* **31**, 1–18 (2012).

61. Mulder, J. A., Berry, R. F., Meffre, S. & Halpin, J. A. The metamorphic sole of the western Tasmanian ophiolite: New insights into the Cambrian tectonic setting of the Gondwana Pacific margin. *Gondwana Res.* **38**, 351–369 (2016).

62. Ranalli, G. *Rheology of the Earth*. (Springer Science & Business Media, 1995).

63. Karato, S. I. & Wu, P. Rheology of the upper mantle: A synthesis. *Science (80-.).* **260**, 771–778 (1993).

64. Bittner, D. & Schmeling, H. Numerical modelling of melting processes and induced diapirism in the lower crust. *Geophys. J. Int.* **123**, 59–70 (1995).

65. Clauser, C. & Huenges, E. Thermal conductivity of rocks and minerals. *Rock Phys. phase relations a Handb. Phys. constants* **3**, 105–126 (1995).

66. Turcotte, D. L. & Schubert, G. Geodynamics. (2002).
